# The modulation of apoptosis by oncogenic viruses

**DOI:** 10.1186/1743-422X-10-182

**Published:** 2013-06-06

**Authors:** Alma Mariana Fuentes-González, Adriana Contreras-Paredes, Joaquín Manzo-Merino, Marcela Lizano

**Affiliations:** 1 Unidad de Investigación Biomédica en Cáncer. Instituto Nacional de Cancerología, México/Instituto de Investigaciones Biomédicas, Universidad Nacional Autónoma de México, Av. San Fernando 22, col. Sección XVI, Tlalpan, C.P. 14080, Mexico City, Mexico

**Keywords:** Apoptosis, Virus, Cancer, Oncogene

## Abstract

Transforming viruses can change a normal cell into a cancer cell during their normal life cycle. Persistent infections with these viruses have been recognized to cause some types of cancer. These viruses have been implicated in the modulation of various biological processes, such as proliferation, differentiation and apoptosis. The study of infections caused by oncogenic viruses had helped in our understanding of several mechanisms that regulate cell growth, as well as the molecular alterations leading to cancer. Therefore, transforming viruses provide models of study that have enabled the advances in cancer research. Viruses with transforming abilities, include different members of the Human Papillomavirus (HPV) family, Hepatitis C virus (HCV), Human T-cell Leukemia virus (HTLV-1), Epstein Barr virus (EBV) and Kaposi’s Sarcoma Herpesvirus (KSHV).

Apoptosis, or programmed cell death, is a tightly regulated process that plays an important role in development and homeostasis. Additionally, it functions as an antiviral defense mechanism. The deregulation of apoptosis has been implicated in the etiology of diverse diseases, including cancer. Oncogenic viruses employ different mechanisms to inhibit the apoptotic process, allowing the propagation of infected and damaged cells. During this process, some viral proteins are able to evade the immune system, while others can directly interact with the caspases involved in apoptotic signaling. In some instances, viral proteins can also promote apoptosis, which may be necessary for an accurate regulation of the initial stages of infection.

## Introduction

Various factors are associated with the development of cancer, including persistent viral infections, which are responsible of 15 to 20% of all neoplastic processes [1].

Studies related to infectious diseases and cancer have contributed significantly to our knowledge of cancer pathogenesis. Several Nobel prizes have been awarded to the researchers in this field [2], including Johannes Andreas Grib Fibiger (1926), for *Spiroptera* carcinoma and its association with gastric tumors in rats; Peyton Rous (1966), for cancer-inducing viruses; David Baltimore, Renato Dulbecco and Howard M. Temin (1975), for the interaction between tumor viruses and the genetic material of the cell; Michael J. Bishop and Harold E. Varmus (1989), for the cellular origin of retroviral oncogenes; and Barry J. Marshall and Robin J. Warren (2005), for the bacterium *Helicobacter pylori* and its role in gastritis and peptic ulcer disease. In 2008 Harald zur Hausen shared the Nobel Prize award for his discovery of human papilloma viruses causing cervical cancer.

Other landmark studies have been of great relevance to the field. For example, in 1991, Harold zur Hausen proposed that a significant fraction of all human cancers worldwide, approximately 1 in 5, are associated with viral infections [3]. In 1910, Peyton Rous studied a cell-free transmissible oncogenic pathogen [4], and in 1932, Shope and Hurst demonstrated the oncogenic activity of a Papillomavirus in domestic rabbits [5]. In 1936, Bittner established the oncogenic role of mouse mammary virus [6], and in 1951, Gross confirmed the viral cause of murine leukemias [7]. In 1964, Epstein and collaborators showed the association of a virus with Burkitt lymphoma [8].

Many researchers have demonstrated the viral etiology of carcinomas of the uterine cervix. In 1974, Beral et al. proposed that cervical cancer was a sexually transmitted disease (STD) [9], and zur Hausen suggested that the Human Papillomavirus (HPV) was the putative oncovirus [10]. It is now indisputable that cervical cancer, penile cancer, some oropharyngeal cancers and other cancers of the anogenital tract are caused by certain strains of HPV. HPV vaccines have demonstrated effectiveness in reducing the incidence of cervical intraepithelial neoplasia [11], confirming the significant contributions of HPV to the development of cervical cancer.

During the same period, Vogel et al. presented preliminary data on the role of Hepatitis B virus (HBV) in liver cancer in Uganda [12], and in subsequent studies, a clear etiological link emerged between HBV and hepatocellular carcinoma [13]. This link was later extended to Hepatitis C virus (HCV) infections. In both cases, establish an association between the virus and tumor development has been complicated, by the long incubation period; the participation of chronic inflammation or cirrhosis in its pathogenesis; and the influence of cofactors, such as dietary and aflatoxins. The HBV vaccine, which was introduced in the last 15 years, has already demonstrated its potential for lowering the risk of hepatocellular carcinoma [14].

The effect of viral proteins in the modulation of cell proliferation and transformation has been widely studied [15,16], and it is now clear that oncogenic viruses may also interfere with the cellular control of apoptosis. Some oncogenic viruses have developed different mechanisms for evading apoptotic signals, mainly via the expression of viral oncogenes. During this process, the deregulation of the cell cycle and apoptotic pathways can lead to changes in the cell that eventually promote cancer development. Some of the mechanisms employed by oncogenic viruses to avoid apoptosis, thus promoting cell transformation, are provided in Table 1[17-28].

**Table 1:** Human viruses related to cancer: viral proteins affecting apoptosis

**Virus**	**Cancer type**	**Protein**	**Mechanism**
Epstein Barr	Burkitt’s lymphoma [17]	EBNA3C	Binds Rb and promotes cell cycle progression
	Hodgkin’s lymphoma [18]	EBNA1	Inhibits p53 induced apoptosis
	Nasopharyngeal carcinoma [19]		
	Gastric carcinoma [20]		
Human Herpesvirus 8 (KSHV)	Kaposi’s sarcoma [21,22]	LANA1 Kaposina	Bind to p53 and inhibit p53-dependent apoptosis
Human Papillomavirus	Cervical cancer [23]	E6	Inhibits p53, Bak, FaDD and procaspase 8
	Oropharyngeal carcinoma [24]	E7	Pleiotropic effects inhibiting and promoting apoptosis
	Anal cancer [25]	E2	Binds and activates caspase 8 (HPV-18);
			Interacts with c-Flip inhibiting its action
Human T-cell leukemia virus type 1 (HTLV)	Adult T-cell leukemia/lymphoma [26]	Tax	Involved in regulation of cell-cycle, apoptosis, cellular transcription, NFkβ, chromatin remodeling
Hepatitis B	Hepatocellular carcinoma [27]	HBx	Activates caspases 3 and 8
Hepatitis C	Hepatocellular carcinoma [28]	Core, NS3 and NS5A	Suppress p53-mediated apoptosis

In many instances, the regulation of apoptotic signaling has been associated with cancer development.

The study of the mechanisms by which viruses regulate apoptosis can contribute to the development of new therapies against infectious diseases and cancer. In this review, we will describe some of the mechanisms used by oncogenic viruses to modulate apoptosis.

### Apoptosis

Apoptosis is a fundamental cellular process required for embryonic development, organogenesis and the elimination of damaged or aged cells during the maintenance of cellular homeostasis [29]. In the physiological context, apoptosis is strictly regulated. When this regulation fails, a number of pathologies may result, such as autoimmune or neurodegenerative diseases and cancer. Apoptosis is a form of cell death that involves a series of ordered events. The first phase is the commitment phase, wherein the cell loses contact with its neighboring cells and presents with modifications of the cytoskeleton, causing a decrease in cell size and changes in cell morphology [30]. During the second phase, the execution phase, there is an increase in intracellular Ca^2+,^ which induces the activation of certain groups of enzymes, such as endonucleases and proteases, such as caspases. Additionally, the chromatin is condensed and fragmented, forming vesicles of different sizes surrounded by a plasma membrane. These vesicles, known as apoptotic bodies, contain parts of the chromatin and cellular organelles [30,31]. The final phase is the termination phase, which involves phagocytosis and the degradation of the apoptotic bodies [30].

Apoptotic death is triggered by different intra- or extracellular stimuli. Intracellular death signals can be induced by cell stress, which promotes the liberation of cytochrome c from the mitochondria [29]. Extracellular stimuli include UV radiation, the depletion of growth factors, and the ligand-mediated activation of death receptors.

### The induction of apoptosis

#### Intrinsic and extrinsic pathways

**Figure 1:**
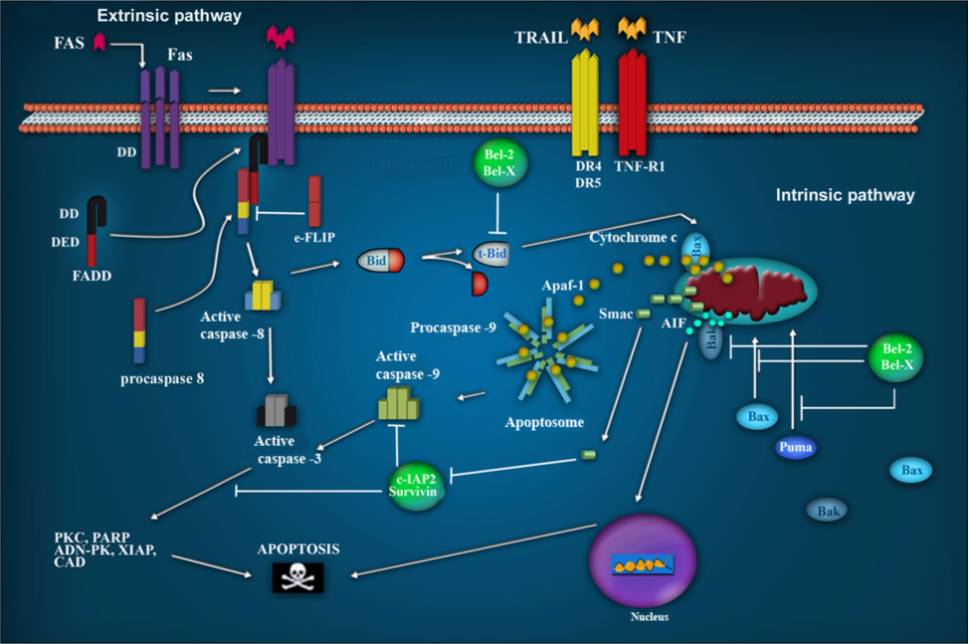
**Apoptotic signaling pathways.** The extrinsic pathway is regulated by membrane receptors. The interaction with their ligands Fas, Trail and TNF, favors their trimerization, inducing the recruitment of FADD through the interaction with their death domains (DDs). The interaction of FADD with procaspase 8 forms a complex called DISC, which favors its oligomerization and auto-cleavage. Active caspase 8 initiates the cascade of effector caspases 3, 6 and 7. In the intrinsic pathway, Bax and Puma are translocated from the cytosol to the mitochondrial membrane as a result of DNA damage, thus provoking the release of cytochrome c. Cytochrome c participates in the formation of the apoptosome, which is involved in DNA degradation. AIF contribute to DNA and nuclear fragmentation.

In mammals, apoptosis is regulated by the activation of two signaling pathways: the extrinsic and the intrinsic pathways. The extrinsic pathway is regulated by membrane death receptors, such as DR4/TRAIL-R1 and DR5/TRAIL-R2. Tumor Necrosis Factor Receptor 1 (TNFR1), and Fas (CD95), are activated by their ligands TRAIL, TNF, and FasL, respectively. The binding of the ligand to its receptor induces the activation of the caspase cascade (Figure 1) [32].

Conversely, the intrinsic pathway is regulated by mitochondrial proteins, that upon activation, cause the release of cytochrome c into the cytoplasm [33,34]. In the cytosol, a complex known as the apoptosome is formed through the binding of Apoptotic Protease Activation Factor 1 (Apaf-1), procaspase 9, and cytochrome c [35-37]. The oligomerization of Apaf-1 activates caspase 9, which, in turn, induces the proteolytic cleavage of other substrates involved in cell death [33-35] (Figure 1).

At the biochemical level, when an inducer triggers a cell death signal in a target cell, the cell death process advances through enzymatic intermediaries, thus directing apoptosis. In both intrinsic and extrinsic pathways, the main effector proteins are the caspases [32,36].

The caspases constitute a family of cysteine proteases that are specific for aspartate. The caspase family members are similar in amino acid sequence, structure, and specificity [36]. Caspases are synthesized as zymogens, and their activation requires specific cleavage at selected aspartate residues. At the initial processing, an inactive caspase is cleaved in a large (p20) and a small (p10) subunits, after which the N-terminal domain is removed to form the catalytically active protease [32,36]. Caspases can be classified into two categories: initiator caspases and executioner caspases. Initiator caspases have a long N-terminal prodomain, which mediates the formation of protein complexes that provide the molecular platform for caspase activation and inhibition [32,36]. Initiator caspases cleave and activate a few specific substrates, including the zymogens of executioner caspases [32,36]. The activated executioner caspases then cleave their respective substrates, which elicit apoptotic cell death, along with its characteristic morphological features, such as membrane blebbing, pyknotic nuclei, cell rounding, and the formation of apoptotic vesicles [36].

#### Inhibitors of caspase activation (IAPs)

A balance between cell proliferation and apoptosis is required to avoid the development of pathologies such as neurodegenerative diseases and cancer. In eukaryotic cells, this balance is maintained mostly by a family of proteins known as IAPs (Inhibitor of apoptosis proteins) [37]. The IAP family is composed of 8 members; however, the best studied proteins in the family are the XIAP (X-linked inhibitor of apoptosis protein), that can directly inhibit the effector caspases (caspases 3 and 7) as well as the initiator caspase 9 [38]. Additionally, XIAP is an ubiquitin ligase; therefore it can indirectly inhibit apoptosis by inducing the degradation of caspases and other pro-apoptotic proteins via the proteasome [39].

Cancer cells express elevated levels of IAPs, which has been associated with chemoresistance, disease progression, and poor prognosis [40]. For example, under normal circumstances, survivin, a member of the IAPs that has been widely associated with the development of cancer, is only expressed in embryonic tissues; however, it has been found to be over-expressed in various tumors [41,42].

#### The role of oncogenic viruses in apoptosis

Although oncogenic viruses have been identified as etiologic agents in the development of different tumors, infection alone is not sufficient to induce cancer development. Most of the people infected by these viruses do not necessarily develop tumors. In those who do develop cancer, many years separate the initial infection and the appearance of a tumor, suggesting that many factors are involved in the transformation process.

A number of viral proteins that are responsible for the oncogenic capability of the virus, interact with elements of the apoptotic signaling pathways and thus inhibit their activities. Some viruses also regulate apoptosis by affecting its inhibitors, such as members of the IAP family and survivin. Conversely, other viral proteins can promote apoptosis, an event that is most likely important for the fine regulation of the initial stages of infection and is not necessarily involved in the transformation process.

#### Human papillomavirus

The main risk factor for the development of cervical cancer is the persistent infection of Human Papillomavirus [43]. Cervical cancer is the second most frequent cancer and the second leading cause of cancer death in women worldwide [44]. High risk HPVs (HR-HPV) refer to HPV types associated with cervical cancer, while Low risk HPVs (LR-HPV) are generally found in benign lesions or low grade cervical dysplasia [45,46].

#### Viral genome and structure

**Figure 2:**
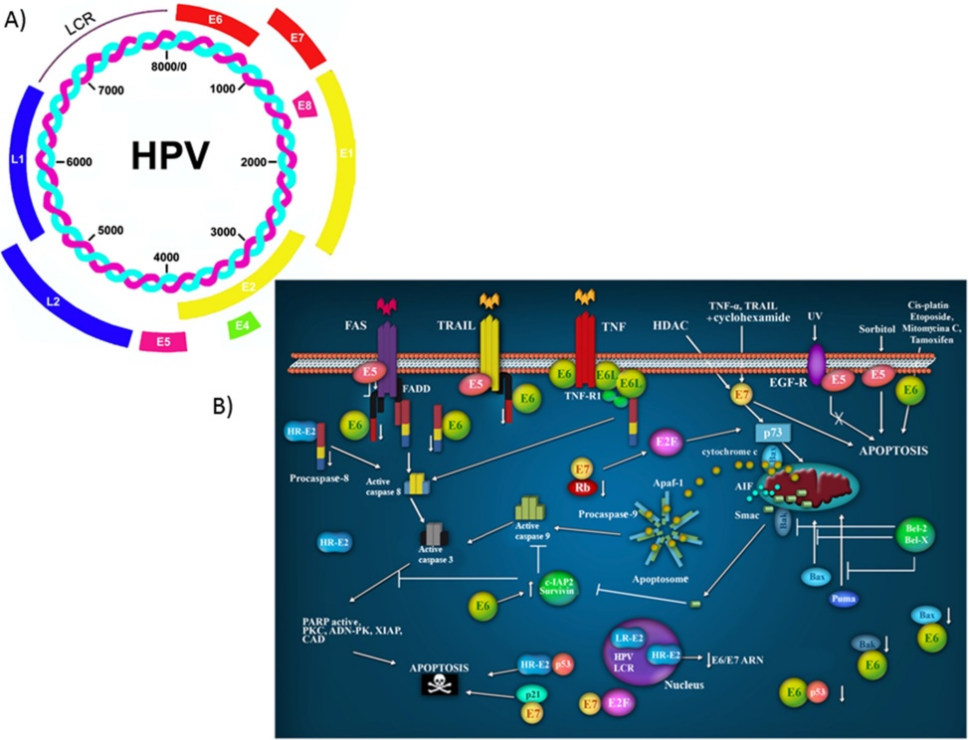
**The HPV proteins involved in apoptotic signaling pathways. A**) The Human Papillomavirus Virus genome. All HPVs have a common genomic organization and encode 8 proteins: E1, E2, E4, E5, E6 and E7 (early) and L1 and L2 (late). **B**) Participation of HPV proteins in apoptotic pathways. E5 impairs the formation of the death-inducing signaling complex triggered by FasL and TRAIL. E6 targets pro-apoptotic proteins such as p53, Bax and Bak for proteolytic degradation; in contrast, E6 can also induce the expression of IAPs. E7 promotes the degradation of the anti-apoptotic protein, pRb, releasing E2F-1. E2 induces apoptosis via the downregulation of E6/E7 mRNA; or direct binding and activation of procaspase 8; or when it binds to p53. Modified from Lagunas-Martínez A. et al. (2010) [49].

HPV is a small virus with a double-stranded DNA genome, that is organized into three distinct regions (Figure 2A). The early expression region (E) encodes proteins implicated in replication and the control of viral transcription (E1 and E2), as well as proteins that are involved in cellular transformation and immortalization (E5, E6 and E7) [47]. The late expression region (L) includes genes involved in capsid formation, L1 and L2. Finally, the region containing the binding sites for numerous factors that control transcription and viral replication is known as the Long Control Region, LCR or URR [48] Figure 2A.

#### Anti-apoptotic effect of HPV viral proteins

Many viruses, including HPV, have developed numerous strategies to block host-mediated apoptosis. The ability of HPV to persist in the host for long periods of time without being eliminated attests to the sophistication of its evasion mechanisms. A growing body of evidence suggests that the oncoproteins of HR-HPVs, E6, E7 and E5, can inhibit death receptor signaling at key points in the pathway (Figure 2B). In doing so, HPV is able to regulate the survival of infected cells to facilitate its replication cycle, thus ensuring the production and spread of its progeny [50]. HPV-positive cervical cancers and cell lines display a differential expression of several caspases and the downregulation of Fas expression, leading to impaired apoptosis [49,51]. Multiple alterations in both caspase expression and activation have been reported in biopsies and cervical cancer-derived cell lines that are HPV positive [49,51].

#### E7 protein

E7 oncoproteins from HR-HPVs can immortalize primary human keratinocytes. These oncoproteins inhibit differentiation and activate cell cycle progression, mainly due to the disruption of the pRb-E2F complex, releasing active E2F and trans-activating several genes involved in DNA synthesis [50]. In addition, E7 is a potent inhibitor of p21CIP1 and p27KIP1 activity, thus bypassing the normal G1 checkpoint control [52]. In addition to its role in cell proliferation and viral replication, E7 has pleiotropic effects on the cellular apoptotic pathways. It has been demonstrated that E7 from HPV-16 induces the degradation of pRb, an anti-apoptotic protein, through the ubiquitin proteasome pathway (Figure 2B) [52], suggesting that E7 might promote apoptosis. The majority of studies suggest that E7 has a pro-apoptotic role. It has been reported that when the HPV-16 E7 oncoprotein is expressed in the lens of transgenic mice, the cells are predisposed to undergo apoptosis that is both dependent on and independent of p53 [53]. Moreover, E7 has been shown to sensitize JD3 mouse lymphoma cells to IFN-alpha-induced apoptosis [54], the co-expression of E7 and p21 induces apoptosis in U2OS osteosarcoma cells [55], and the overexpression of E7 in genital-derived keratinocytes induces spontaneous cell death and sensitizes the cells to TNF-mediated apoptosis [56]. However, in some studies, E7 appears to be anti-apoptotic. Yuan *et al.* suggested that E7 can inhibit TNF-mediated apoptosis in keratinocytes by up-regulating the expression of the inhibitor of apoptosis protein, c-IAP2, and an antiapoptotic protein [57]. In another study, it was reported that the expression of E7 in fibroblasts delayed Fas-mediated apoptosis and prevented TNF-mediated apoptosis by suppressing caspase-8 activation [58].

The pleiotropic effects of both E6 and E7 on apoptosis is indicative of their important role in immune evasion and underscores the complexity of HPV-host interactions.

#### E6 protein

The E6 protein binds to numerous cellular targets implicated in proliferation and apoptosis. One of the functions of the HR-HPV E6 oncoproteins is the proteolytic inactivation of certain pro-apoptotic proteins such as p53 [59], Bak [60], FADD [61], procaspase-8 [62] and c-myc [63], through the ubiquitin proteasome pathway (Figure 2B).

Bak and myc were the first apoptosis-related targets of E6 to be identified. Thomas and Banks found that E6 inhibits Bak-mediated apoptosis by directly binding to Bak, an interaction that is conserved from HR- to LR-HPVs [64]. In laryngeal cells, E6 was found to inhibit TNF-mediated apoptosis by reducing the expression of Bak, without significantly affecting the expression of caspase-3 and caspase-8. As in the case with p53, both Bak and myc are ubiquitinated by E6AP, are able to bind to E6 and are degraded in the ubiquitin-proteasome pathway [64].

#### E5 protein

Recent studies have shown that the E5 protein inhibits apoptosis mediated by the TRAIL and Fas receptors (Figure 2B). E5 reduces the affinity of Fas for its ligand. It blocks the TRAIL-mediated apoptotic signaling pathway by preventing the formation of the TRAIL-DISC complex and inhibits the proteolysis of caspases 8 and 3, as well as of PARP [65].

E5 also protects tumor cells from apoptosis induced by UV-irradiation by enhancing the PI3K–Akt and ERK1/2 MAP kinase signaling pathways [66]. In addition, the HPV-16 E5 protein inhibits hydrogen peroxide-induced apoptosis by stimulating the proteosomal degradation of Bax. In contrast, E5 was also reported to sensitize human keratinocytes to apoptosis induced by osmotic stress [66]. However, this effect may be due to cell membrane modifications caused by the highly hydrophobic E5 protein. By modulating apoptosis, HPV 16 E5 allows HPV 16-infected cervical cells to evade apoptosis induced by physical or chemical stimuli. In addition, HPV 16 E5 may protect infected cells from apoptotic stimuli derived from immune effector cells by impairing FasL- and TRAIL-mediated apoptosis, thus contributing to the evasion of host immunosurveillance. All these activities may ultimately lead to cervical carcinogenesis.

#### The pro-apoptotic effect of viral proteins

Viral infections can also promote pro-apoptotic processes, and these opposing effects on apoptosis can be mediated by the same proteins. For example, E6 and E7, which can inhibit apoptosis, can also promote it. The viral apoptotic effect is better understood during the establishment of an infection. The life cycle of HR-HPV involves the fine regulation of the expression of viral oncogenes that will allow the cellular differentiation necessary to produce viral particles.

Moody *et al*. [67] reported that HPV proteins activate rather than suppress caspases, and this could be a necessary condition for the productive HPV life cycle. The authors observed that the treatment of HPV-31-positive cells with caspase inhibitors significantly reduced viral genome amplification. The identification of a caspase 3/7 cleavage site (^46^ DXXD ^49^) in the viral replication protein E1, which is conserved in all genital HPVs, suggests that this motif provides an important function in the differentiation-dependent life cycle of papillomaviruses [67]. It is possible that the expression of antiapoptotic proteins, coupled with a low level of caspase activation, may be important in providing the balance between cell viability and cell death upon differentiation.

#### Protein E2

The viral E2 protein plays a critical role in the HPV life cycle due to its ability to regulate viral DNA replication and the transcription of E6 and E7 oncogenes [68]. The integration of the viral DNA into the cellular genome is considered a key element in the transformation process. Viral episome rupture during integration frequently occurs in a zone that limits E2 expression. Therefore, it is probable that the effects of the full-length E2 will occur preferentially during the initial stages of infection.

The direct induction of apoptosis by E2, independently of E6 and E7, was first demonstrated in 1997 by Frattini *et al*. [69], who observed the death of human foreskin keratinocytes, when they were infected with adenovirus expressing E2 from HPV31. Desaintes *et al*. [70], showed that in HeLa cells, apoptosis was induced only by the full-length E2 protein from HPV18, and not when the transactivation domain of E2 was deleted. As both proteins can repress the transcription of E6 and E7, this result indicated that apoptosis does not occur through the repression of the viral oncogenes.

Some studies have shown that E2 can induce apoptosis in HPV negative cell lines. Furthermore, this protein binds to and activates pro caspase 8, through its transactivation domain, overcoming the need for adaptor proteins involved in the classical extrinsic pathway that is Fas-dependent [71] (Figure 2B).

The involvement of caspase 8 in apoptosis induced by E2 was also demonstrated in HPV16, in which E2 directly interacts with c-FLIP [72].

Because E2 is expressed in the intermediate differentiated layers of the HPV infected lesions, it is possible that in vivo, the modulation of caspase 8 by E2 might play a role in the formation of warts, via an as yet unknown mechanism [73].

The role of p53 in E2- induced apoptosis is controversial. E2 induces apoptosis in HPV positive- and negative-cell lines through both p53 dependent and p53 independent mechanisms [71,74]. It is worth mentioning that E2 proteins from the low-risk HPV6 and HPV11 cannot induce apoptosis, which could be due to their cellular localization, because the E2 proteins of low-risk HPVs are located only in the nucleus, whereas those of HR-HPVs are localized in the both nucleus and cytoplasm [74].

Even the role of E2 in apoptotic induction in HPV life cycle is not yet understood, this effect could be related to the activation of E1 during viral genome replication. E2 could also be inducing apoptosis in those cells that do not allow the virus to properly complete the viral cycle.

#### Hepatitis viruses

Liver cancer or hepatocellular carcinoma (HCC) is the third leading cause of cancer related deaths in the world [75]. It is the fifth most common cancer in men and the eighth in women. The Hepatitis virus is the main etiologic agent of HCC [76]. The Hepatitis viruses are the most common infections that affect the liver. To date, 5 responsible agents for hepatitis have been identified and are characterized as follows: Hepatitis A virus (HAV), B (HBV), C (HCV), D (HDV), and Hepatitis E virus (HEV). HBV and HCV are responsible for 70% of hepatocellular carcinoma, of which 60% are caused by HCV [77,78]. This phenomenon can be explained by certain biological and clinical characteristics of HCV that favor hepatocarcinogenesis, such as the high capacity of HCV to induce a chronic infection. In contrast, after 10 years of infection, HBV only induces chronic cirrhosis in a small percentage of patients (5-10%), while the percentage of patients who develop this disease as a consequence of HCV infection is 55-60% [79].

#### Viral genome and structure

**Figure 3:**
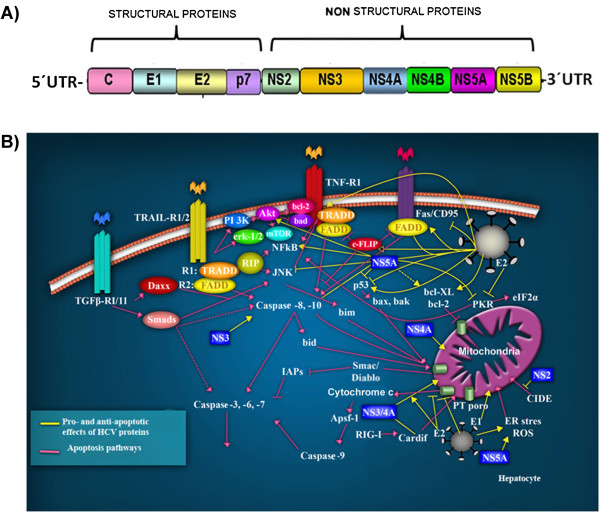
**The Hepatitis C virus and apoptotic signaling pathways. A**) The Hepatitis C virus genome. A single open reading frame encodes four structural proteins and six nonstructural proteins. **B**) HCV-infected hepatocytes are recognized by the immune cells, that promote apoptosis via the death receptor ligands, TRAIL, TNFα, CD95 ligand, and TGF-β, as well as granzyme B/perforin (**Pink lines**). Ligand-induced apoptosis activates caspase-8, whereas activation of caspase-9 occurs via the mitochondrial permeability transition (PT) pore, triggering the activation of caspases cascade and the irreversible induction of apoptosis. For virtually all HCV proteins, pro- and anti-apoptotic effects have been described (**Yellow lines**). The structural (core C, E1 and E2) and nonstructural (NS2, NS3, NS4A and NS5A) proteins participate in the extrinsic and intrinsic apoptotic pathways. Modified from Fischer R. et al. (2007) [92].

HCV belongs to the *Flaviviridae* family [80]. Its genome comprises a single strand of DNA which encodes a single 3000 bp open reading frame (ORF), flanked by untranslated regions (UTR) at the 5′ and 3′ ends (Figure 3A) [81]. The ORF encodes a polyprotein that is processed to produce three structural proteins, core C, E1 and E2, a small integral protein p7, and six nonstructural (NS) proteins NS2, NS3, NS4A, NS4B, NS5A, and NS5B. The structural proteins are found in the N-terminal region, while the nonstructural proteins are encoded by the C-terminus (Figure 3A) [82]. The main functions of these viral proteins are summarized in Table 2[83-91] Figure 3A.

**Table 2:** Functions of HCV viral proteins

**Structural proteins**	**Functions**
Core	Forms the nucleocapsid and participates in signaling pathways that control cellular proliferation and apoptosis [83].
E1 and E2	Glycoproteins that make up the viral envelope, they bind to the receptor on the host cell to facilitate viral entry [84].
P7	Creates hydrophobic pores with ionic channel activity. Fundamental in the assembly and release of viral particles [85].
**Nonstructural proteins**	
NS2	Forms a catalytic complex with NS3 to cleave the NS2-NS3 junction. Important for the production of infectious viruses [86].
NS3	Serine protease that cleaves the junctions between NS3/NS4A, NS4A/NS4B, NS4B/NS5A and NS5A/NS5B. Contains a helicase domain implicated in viral replication. Cofactor NS4A [87].
NS4A	Important cofactor for NS3 activity participates in host innate immune response evasion and regulates viral transcription [88].
NS4B	Induces formation of membranous web specialized sites where RNA replication takes place [89].
NS5A	Binds viral RNA participating in viral replication; promotes particle assembly [90].
NS5B	Is the RNA polymerase responsible for replication of the viral genome [91].

#### Apoptotic processes induced by HCV infection

The induction of apoptosis is a mechanism used by hepatocytes to defend against HCV infection. The immune response is mediated mainly by macrophages and natural killer (NK) cells, which can directly cause the death of the infected cells [93]. Additionally, this process can be mediated by the receptors and ligands of the Tumor Necrosis Factor family, specifically, the TNFα/1 receptors, CD95/CD95 ligands, and TRAIL receptors 1 and 2 (Figure 3B) [93]. The binding of the ligands to the death receptors results in the activation of caspase 8 which in turn, activates two signaling pathways. The first pathway involves the proteolytic cleavage of Bid, the release of mitochondrial cytochrome c, the activation of caspase 9 [94], and the effector caspases 3, 6 and 7. In the second signaling pathway, caspase 8 directly activates the effector caspases. In this case, apoptosis is also regulated by inhibitors, such as survivin and c-FLIP, which can block caspase activity [95]. HCV viral proteins have the ability to inhibit host induced apoptosis, fact that could allow the establishment of a persistent infection.

#### The core protein

It has been demonstrated that the core protein of HCV has both pro-apoptotic and anti-apoptotic functions. This protein can inhibit CD95 receptors and TNFα induced apoptosis by inhibiting the liberation of cytochrome c and, thus, by activating caspases 9, 3 and 7 (Figure 3B) [96]. Additionally, the direct binding of the core protein to the cytoplasmic domains of the CD95 and TNFα receptors has been reported to induce a pro-apoptotic effect by altering mitochondrial function. Specifically, this effect induces the production of reactive oxygen species, causing a change in mitochondrial membrane potential, which permits the release of cytochrome c [97]. Furthermore, it has been postulated that this protein can bind to death domains, such as FADD and to the c-FLIP inhibitor, resulting in an anti-apoptotic effect [98]. Many studies have indicated that the core protein can modulate p53 in a positive or negative manner [99,100].

HCV can also induce apoptosis through the interaction of NS5A with the protein kinase R (PKR), the kinase regulated by double-stranded RNA (dsRNA). PKR has different functions, such as the evasion of the antiviral action of interferon and the induction of apoptosis. This kinase catalyzes the phosphorylation of the transcription factor eIF-2, leading to the inhibition of anti-apoptotic protein synthesis during viral infection [101] (Figure 3B). In turn, PKR is activated via binding to the NS5A viral protein.

#### E1 and E2 proteins

As is the case for other oncogenic viruses, is clear that Hepatitis C has a dual role in regulating apoptosis. For instance, HCV E1 and E2 proteins, which mediate the binding and entry of HCV into the host cell, are capable of inhibiting Fas-mediated apoptosis by repressing the activation of caspase-8 and the release of cytochrome c from the mitochondria [102]. However, these structural proteins increase the expression of FasL and the ability of hepatocytes to induce apoptosis in activated CD4+ and CD8+ T cells, which may contribute to the persistence of HCV [103,104].

#### Nonstructural proteins

**Figure 4:**
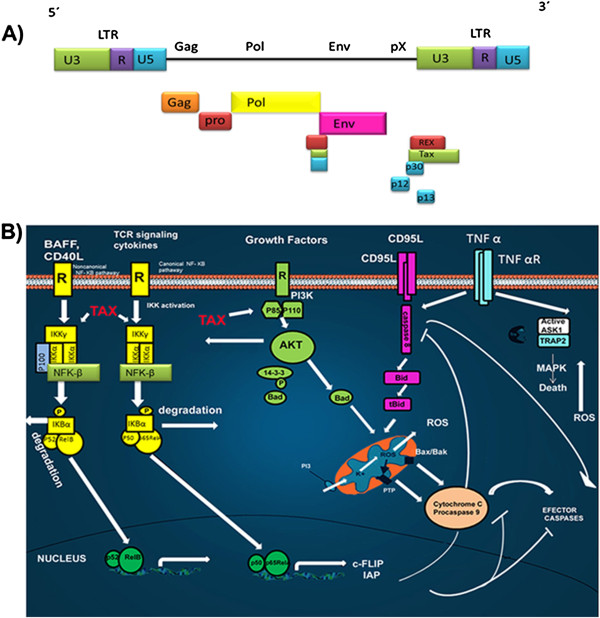
**HTLV-1 and apoptotic signaling pathways. A**) The HTLV-1 viral genome. The genome consists of a single- positive strand of RNA. The long terminal repeats (LTRs) flank the ORF (boxes) of the structural (orange, red, yellow and pink) and the nonstructural (blue, green) viral proteins. **B**) Main death pathways controlled by HTLV-1 proteins. In infected cells receptor-mediated death (through CD95/Fas) is inhibited by a Tax/NF-kB-mediated upregulation of c-FLIP and IAPs. The PI3K-AKT pathway, also activated by Tax, acts by inhibiting the pro-apoptotic protein Bad and by activating the NF-kB pathway. The accessory proteins, p12 and p13, regulate Bcl-2 and caspases 3 and 9, and also ROS production by mitochondria.

Figure 4 shows the roles played by HCV nonstructural proteins in the apoptotic pathways. The processing of nonstructural proteins involves the formation of autocatalytic protein complexes. NS2 is a transmembrane protein, found in the endoplasmic reticulum. It binds to and activates cell death-inducing DNA fragmentation factor (DFFA)-like effector b, (CIDE-B), which is a key inducer of the extrinsic apoptotic pathway [105].

The NS3 protein promotes the degradation of Cardif, a protein that translocates to the mitochondrial membrane and activates the intrinsic pathway [106]. When it associates with the NS4A cofactor protein, a complex is formed. This complex localizes in the mitochondria and participates in the release of cytochrome c and the activation of caspase 8 [107]. The functions of NS5A are not well defined yet; but it is thought to interfere with the response to IFN and may participate in viral replication. With respect to its role in apoptosis, this protein has sequences homologous to bcl-2 and binds to FKBP38, increasing the anti-apoptotic effect of Bcl-2. Conversely, it has been demonstrated that NS5A inhibits the pro-apoptotic activity of Bax in hepatocytes cells [108]. The anti-apoptotic effect of NS5A is also mediated by the recruitment of p53 in the cytoplasm, the activation of STAT3, and the increase in the expression of Bcl-XL and p21.

The impact of the induction of apoptosis in chronic HCV infection not well understood. Almost for each of the viral protein studied, according to the experimental model, pro-apototic and anti-apoptotic effects have been identified. The modulation of apoptosis by HCV proteins is an important issue to study in order to understand its role in acute HCV infection and persistence.

#### Human t-cell leukemia virus type 1 (human t-lymphotropic virus type 1)

Currently, there are close to 20 million people infected with the Human T-Cell Leukemia virus type 1 (HTLV-1) worldwide and between 3 to 5% of these individuals develop diseases related to this infection [109].

HTLV-1 is a member of the *Retroviridae* family, which is in the *Oncovirus* subfamily. It is a RNA retrovirus that is involved in carcinogenic processes due to its participation in malignant adult T-cell leukemia. Additionally, it is involved in the development of a subacute myelopathy, termed HTLV-1 associated myelopathy [110].

#### Viral genome and structure

HTLV-1 mainly infects CD4+ T-lymphocytes; once the infection has been established, it can remain integrated in the host in the form of a provirus. HTLV-1 has a relatively small genome of 9 kb, comprising the structural and enzymatic genes gag, pro, pol, and env, which are flanked by two terminal regions of repeated sequences (LTRs) (Figure 4A) [108]. The long terminal repeat (LTR) region is subdivided into three regions, U3, R and U5 and contains cis-active elements that are essential for the transcription and expression of viral genes. The pX region contains four open reading frames (ORFs), that encode the accessory proteins (p12^I^, p13^II^, p30^II^), the posttranscriptional regulator REX (ORF III) and the transactivator Tax (ORF IV) [111]. The regulatory proteins Tax and HBZ play a particularly important role in viral persistence and pathogenesis.

#### Role of Tax in apoptosis

Tax is a nuclear protein encoded by HTLV-1 that has been implicated in viral replication, because it is a transcriptional activator of the LTR. This protein participates in infection, cell proliferation and cell survival [112]. Tax can also activate transcription factors, such as: NF-κB, CREB, SRF, and AP-1.

Tax suppresses a wide wide range of pro-apoptotic factors and induces the expression of apoptosis inhibitors. Tax regulates important signaling pathways, such as the nuclear factor of kappa light polypeptide gene enhancer in B-cells (NF-κB), and Akt, both anti-apoptotic proteins which are currently being studied as possible targets for the treatment of adult T-cell leukemia/lymphoma (ATLL) (Figure 4B) [113]. NF-κB is regulated by a family of inhibitors, IkappaB, that retain NF-κB in the cytoplasm, thus preventing its function. The phosphorylation of IkappaB inhibitors by the IKK complex leads to their ubiquitination and degradation, thus activating NF-κB [114]. This effect induces the transcription of a series of anti-apoptotic proteins, such as the Bcl-xL [115] and expression of IAP proteins [116]. Tax activates IKK [117] and can form complexes with the IKKα / IKKγ proteins, thus activating NF-κB [118]. Additionally, Tax can directly regulate the transcription of CBP/p300, a transcriptional coactivator of NF-κB [117-119].

Tax also modulates the signaling pathway regulated by Akt, which is constitutively active in the majority of patients with ATLL [120]. Akt induces the activation of transcription factors, such as AP-1 and β-catenin [121], leading to expression of Bcl-xL, the repression of p53, and overall cell survival.

In addition to the structural proteins, HTVL-1 encodes two accessory proteins, p12 and p13, that have been implicated in the regulation of Bcl-2 family members and caspase 3 and 9 (Figure 4B) [122].

#### The Epstein-barr virus

The Epstein-Barr virus (EBV) belongs to the gamma-1 subfamily of the herpes virus, also called *lymphocriptovirus* (LCV). The LCVs only affect primates and EBV is the only member that infects humans. EBV was initially isolated from Burkitt lymphoma (BL) cells [123]. After primary infection, this virus can establish long-term latent infections in B-lymphocytes. EBV has been associated with a number of lymphoid and solid tumors in both immunocompetent and immunocompromised individuals.

#### Viral genome and structure

**Figure 5:**
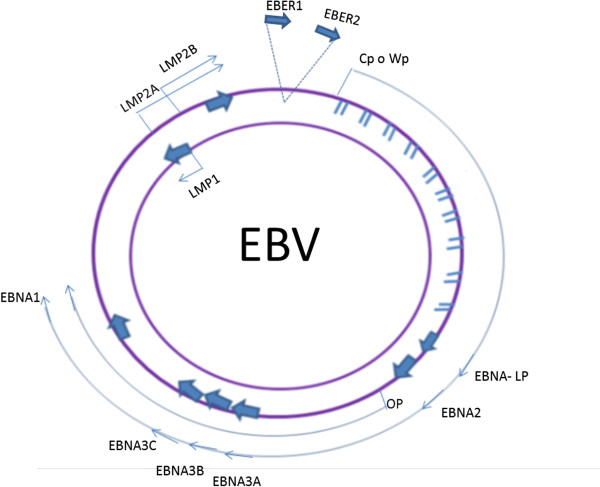
**EBV genome.** Location and transcription of the EBV latent genes on the double-stranded viral DNA episome. The bottom arrows represent coding exons for each of the latent proteins; the latent proteins include the six nuclear antigens (EBNAs 1, 2, 3A, 3B and 3C, and EBNA-LP) and the three latent membrane proteins (LMPs 1, 2A, 2B). EBNA-LP is transcribed from variable numbers of repetitive exons. The arrows with the discontinuous lines close together at the top represent the highly transcribed nonpolyadenylated RNAs EBER1 and EBER2; their transcription is a consistent feature of latent EBV infection. EBNAs are transcribed from either the Cp or Wp promoter.

The Epstein-Barr virus has a linear, double-stranded DNA genome of approximately 184 kb that is wrapped inside a protein capsid (Figure 5). Its DNA contains a short U_S_ and a long U_L_ domain that encode the majority of its viral proteins, the internal region, IR1, and the terminal tandem repeat region, TR. When the virus infects a cell, which typically only requires a single virion, the ends of the linear genome bind to each other and persist as episomal DNA [124]. During the latent phase, there is no production of EBV virus and only a small number of viral genes are expressed. These genes affect the normal B cell growth mechanisms, leading to the immortalization of the cells [125]. The latent infection of immortalized B cells is associated with six nuclear antigens, EBNA1, EBNA2, EBNA3A, EBNA3B, EBNA3C and the leader protein EBNA-LP; three membrane proteins, LMP-1, -2A and -2B; two small nuclear RNAs, EBER1 and EBER2; and transcripts from the BART region, which encodes the majority of the EBV micro RNAs (miRNAs) [126]. The expression of the complete repertoire of viral latent genes is referred to as Latency III [127,128].

The BZLF1 and BRLF1 proteins are key mediators of the transition from the latent cycle to the lytic cycle transition. These proteins are transactivators for other genes related to the lytic cycle and induce the expression of the viral DNA polymerase. To induce the replication, approximately 80 viral proteins are expressed during the lytic phase, including transcriptional activators, DNA replication factors, and structural proteins, such as the antigens that form the viral capsid.

#### EBV and apoptosis

The fact that EBV positive BL tumor cells present the virus in a latent form strongly suggests that EBV is essential for the survival of BL cells *in vivo*. Even though the virus can be eliminated from BL cells in culture through continuous passages, the direct elimination of EBV from these cells induces apoptosis [129]. EBNA1, the EBERs, and the viral miRNAs have all been proposed to be involved in BL cell proliferation and/or resistance to apoptosis, thus conferring a selective advantage to neoplastic cells. There is evidence that EBNA1 has an anti-apoptotic effect in BL cells [124], but the mechanism has yet to be elucidated. Some studies suggest have suggested that the EBERs and EBNA1 are sufficient to promote the malignant growth of BL cells *in vivo*, even in the absence of any other latent phase EBV proteins [129,130].

PKR is a central effector of many apoptotic and stress signaling pathways, and is activated through diverse stimuli, including dsRNA. EBER1 has been shown to be an inhibitor of PKR [131]. The EBERs are dsRNA molecules that have the ability to inhibit PKR activity by binding to it, thus preventing further interactions with other dsRNA molecules and precluding the induction of antiviral and apoptotic pathways. The role of EBER in PKR inhibition during tumorigenesis has not been elucidated. However, the tumorigenic potential of cells that express inactive PKR has been clearly documented [132]. In addition to inhibiting PKR, EBERs have been implicated in apoptosis resistance via the alteration of the expression of the central anti-apoptotic factor, Bcl-2. Initial studies have shown that BL clones expressing EBER also have increased expression of Bcl-2 [133].

Moreover, during the EBV infectious cycle, the viral protein LMP1 has been proposed to mimic the signaling induced by CD40 by providing erroneous survival signals in infected B cells within the germinal center [134]. LMP1 can contribute to neoplastic transformation and to tumor progression by modulating the TNF receptor pathway, through its interaction with the CTAR1 and CTAR2 domains in a ligand-independent manner [135]. In turn, these domains interact with the factors associated with TNF-R (TRAFs) and the death domains coupled with TNF-R (TRADDs) [136]. The association of LMP1 with the TRAF and TRADD molecules activates a signaling cascade that results in the constitutive activation of the JNK, NFKB and PI3K pathways. The activation of these key-signaling pathways increases cellular growth and promotes survival through the induction of anti-apoptotic factors, including Bcl-2 and A20.

#### Kaposi’s sarcoma Herpesvirus

Kaposi’s sarcoma (KS) is a malignant, multifocal systemic disease that originates from the vascular endothelium. The disease has a variable clinical course and most frequently manifests as skin lesions. Different clinical forms can be distinguished, including the so-called classic Kaposi’s sarcoma, which results from immunosuppression and often occurs in organ transplant recipients or after long-term cortisone treatment; the endemic African Kaposi’s sarcoma; and the epidemic HIV-associated Kaposi’s sarcoma. KS is among the most common malignancies occurring in the HIV-infected patients. Although the incidence of AIDS-associated KS has declined since the implementation of highly active antiretroviral treatment (HAART), up to 50% of patients with AIDS-KS never achieve total remission [137]. All types of Kaposi’s sarcoma are due to the infection with Kaposi’s sarcoma-associated herpesvirus (KSHV), also known as Human Herpesvirus 8 (HHV-8) [138]. While its routes of transmission are not completely understood, important known routes are sexual transmission, saliva, blood or organ transplantation [139]. In addition to KS, KSHV has been associated with lymphoproliferative disorders, including multicentric Castleman’s disease (MCD), plasmablastic lymphoma, and primary effusion lymphoma (PEL) [140].

KSHV infects endothelial cells or circulating endothelial and/or hematopoietic progenitors [141]. Its oncogenicity is supported by the numerous pro-angiogenic molecules that are induced following the infection of endothelial cells, including the VEGF-VEGFR family, cyclooxygenase 2 (COX2) and angiogenin [142]. However, in the general population, KSHV infection rarely leads to KS, indicating the need of cofactors, such as immunosuppression, in order for a tumor to be induced.

#### The KSHV genome

The KSHV genome is a linear, double-stranded DNA of approximately 165 to 170 kb in length [143]. During latency, it may also exist in a circular, episomal form in the host nucleus [144]. Among the viruses that infect humans, KSHV is most closely related to the gammaherpesvirus, Epstein Barr (EBV).

KSHV encodes 87 open reading frames (ORFs) and at least 17 microRNAs, 14 of which co-express as a cluster. KSHV has at least 14 ORFs that encode cellular orthologues that play important roles in controlling the cell cycle and cell signaling [145].

The life cycle of all herpesviruses includes prolonged latent and lytic phases. Reactivation occurs when the promoter of ORF50 is activated and the replication and transcription activator RTA is expressed, which is the main regulator of the lytic replication program [145]. During the latent phase, a subset of genes are expressed, such as the latency-associated nuclear antigen (LANA), vCyclin, vFLIP, kaposins and KSHV-encoded 17 miRNAs, which are derived from the processing of 12 pre-miRNAs [146]. These genes are required for viral episome maintenance, host cell survival, and the suppression of lytic gene activation [147]. These protein increase proliferative signals, decrease apoptosis and induce the activation proangiogenic and inflammatory signals, as well as limitless replicative potential.

### The role of KSHV in apoptosis

#### Latent phase proteins

**Figure 6:**
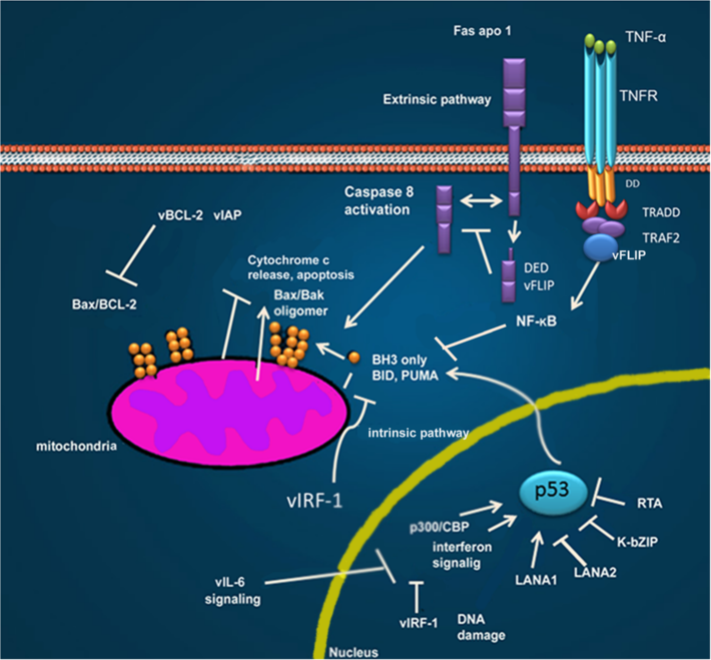
**Different KSHV proteins inhibit intrinsic and extrinsic apoptotic pathways.** vFLIP directly binds to death effector domains, or to the TRAF complex, inhibiting activated-Fas signaling, or activating NF-kB. Both vIAP and vBCL-2 act at the mitochondria to stabilize the mitochondrial membrane and inhibit the activating effects of BH3-only pro-apoptotic molecules.vCyclin LANA1, LANA2, K-bZIP and RTA inhibit p53-induced apoptosis either through direct binding or through inhibition of the p300/CBP coactivator used in p53 transcriptional signaling. vIRFbinds and inhibits pro-death activities of proteins Bid and Bim. Modified from Moore P.S. et al. (2007) [164].

The multifunctional protein, LANA, maintains the viral episome and can also interfere with important cellular processes. The main functions of KSHV latent proteins are exposed in Table 3. LANA is considered to be an oncogenic protein due to its ability to dysregulate tumor suppressor pathways associated with p53 and pRb and to transform primary rat embryo fibroblasts in cooperation with the cellular oncogene H-ras [148]. In addition, this protein has been shown to deregulate Wnt signaling by altering the subcellular distribution of glycogen synthase kinase 3 (GSK-3), a negative regulator of β-catenin [149]. LANA modulates apoptosis by direct binding to p53 (Figure 6). It also associates with different host cell proteins, including chromatin-associated proteins, which are involved in the epigenetic silencing of TGFβ expression. These associations have antiproliferative and apoptotic effects on epithelial, endothelial, and hematopoietic cell lineages [150] Table 3.

**Table 3:** Functions of KSHV viral proteins

**Latent phase proteins**	**Functions**
LANA 1,2	Establish and maintain the latency in KSHV infected cells, bind directly to p53 and pRb [148].
Kaposin A, B	Induce the expression of growth factor receptors, possible transformation activity [151].
vcyclin	Forms a complex with CDK-6 to inactivate the Rb protein, promoting cell cycle progression and proliferation [152]. Induces apoptosis independent of p53 [153].
vFLIP	Blocks caspase 8 activation [154], potent activator of NFκB [154].
**Lytic phase proteins**	
ORF50 (RTA)	Regulates the lytic replication [155].
K1	Activator of the molecules that mimic signaling via the B cell antigen receptor [156].
K8	Regulates lytic-cycle DNA replication [157].
K3, K5	Mediate the down regulation of several immunomodulatory proteins, including CD86, intercellular adhesion molecule 1 (ICAM-1; CD54), and IFN-R [158].
vIL-6	Induces angiogenesis and tumorigenesis by regulating PI3K/PTEN/AKT/GSK-3β signaling pathway [159].
vIRF-1	Binds and inhibits pro-death activities of proteins Bid and Bim [160].
vMIPs	Binds to chemokine receptors and induce angiogenesis [161].
vGPCR	Transformation activity; promotes the secretion the growth factors, such as VEGF, bFGF, IL-8, and IL-6 [162].
vBcl-2	Inhibits apoptosis [163].

vCyclin (viral homolog of cellular cyclin D) is a constitutive activator of cyclin dependent kinase 6 (CDK6). The expression of vCyclin and the formation of the complex, vCyclin/CDK6, leads to defects in cytokinesis, which result in polyploidy and the activation of p53 [152]. However, in the absence of functional p53, cells survive, exposing the oncogenic role of vCyclin. Substrates of the vCyclin/CDK6 complex include pRb and p27 [153]. As such, vCyclin efficiently accelerates cell-cycle progression, even in the presence of CDK inhibitors. In contrast, it has been demonstrated that the expression of vCyclin in cells with increased levels of CDK6 triggers apoptosis independently of p53 and pRb. These findings suggest that vCyclin may have both growth-promoting and apoptotic functions in the development of Kaposi’s sarcoma.

vFLIP (viral FLICE inhibitory proteins) is a small polypeptide composed of two tandem death effector domains (DEDs). The protein is homologous to the cellular FLIP proteins, which are also called FLICE, and blocks the signaling of caspase-8 (Figure 6). This protein could be recruited to DISC through the interaction of its tandem DEDs with DED. As such, FLIP excludes procaspase 8 from the DISC complex [154].

Several KSHV miRNAs have also been shown to modulate host gene expression, suggesting some roles for the miRNAs in the pathogenesis of malignancies induced by KSHV [165]. The target of miR-K5 is the Bcl2 associated factor, BCLAF1, which promotes apoptosis [166]. MiR-K1 targets IκBα, an inhibitor of NF-κB, which inhibits the activation of lytic viral promoters [167].

#### Lytic phase proteins

The aberrant expression of the ORF50 protein is required for the initiation of the lytic phase and the expression of many KSHV-encoded lytic genes, such as K1, K3, and K5; viral macrophage inflammatory proteins (vMIPs); K12; viral G-protein-coupled receptor (vGPCR); viral dihydrofolate reductase (vDHFR); DNA replication factors; and thymidylate synthase [168].

Other lytic proteins that are important in cellular transformation are the viral orthologues of cellular proteins such as viral interleukin-6 (vIL-6), vBCL-2, vIRF and vCCLs, whose functions are summarized in Table 3. vBCL-2 inhibits apoptosis through the inhibition of pro-apoptotic BH3 domain-containing proteins (Figure 6) [169,170]; while vIRF1 inhibits p53-induced apoptosis through its interaction with the central DNA-binding domain of p53 and with the upstream ATM kinase [170].

K1, which is the first ORF of KSHV, inhibits apoptosis by inducing the release of growth factors such as VEFG, leading to the subsequent activation of the PI-3 K-AKT pathway. Prior to cell lysis, the inhibition of apoptosis by lytic proteins could also contribute to cell transformation, viral replication and virion production and assembly [171].

## Conclusions

With the acceptance that tumor viruses account for a substantial fraction of human cancers, tumor virology has evolved from a niche area of research to a central and active field of cancer research. The recent development of powerful new virus detection methods may further extend the spectrum of virus-associated cancers in the future. Cancers exhibiting epidemiological features that are compatible with an infectious cause and cancers that are linked to immunosuppression, are particularly interesting candidates to screen, with the goal of identifying new tumor viruses. Tumor viruses represent promising targets for specific preventive and therapeutic anticancer strategies, as evidenced by the success of the HBV and HPV vaccines. These findings should further motivate research on improved or novel prophylactic vaccines that may protect against other tumor viruses. The deeper understanding of the biology of oncogenic viruses and the defense mechanisms of the host should also facilitate the development of specific therapeutic approaches, because viruses represent targets that are unique to diseased cells.

Successful viral replication requires not only the efficient production and spread of viral progeny, but also the evasion of host defense mechanisms that limit viral replication by killing the infected cells. In addition to inducing immune and inflammatory responses, most viruses encode proteins that interact with the biochemical pathways regulating apoptosis of the infected cell. For some viruses, the inhibition of apoptosis seems to be essential for the maintenance of viral latency. For other viruses, the carefully choreographed induction of apoptosis during infection may represent the basis for cytotoxicity and be an important outlet for the dissemination of virus progeny. For non-lytic virus, pro-apoptotic effects could be implicated in a properly completion of the viral cycle. As these processes are understood in greater detail, the opportunities for the development of new drugs to combat clinically important viruses will almost certainly arise. Such drugs could promote the early death of infected cells, inhibit virus release or, in the case of latent viruses, manipulate the latency switch to minimize the effects of infection.

As the infection mechanisms of oncogenic viruses are better characterized, remarkable insights into the molecular biology of apoptosis will be forthcoming.

## Competing interests

The authors declare that they have no competing interests.

## Authors’ contributions

AMFG prepared the Human Papillomavirus chapter, ACP prepared the Hepatitis Viruses chapter and participated in the Kaposi’s Sarcoma Herpesvirus chapter, JMM prepared the HTLV-1 chapter, ML participated in the design and coordination of the manuscript and prepared the Epstein-Barr Virus and the Kaposi’s Sarcoma Herpesvirus chapters. All authors read and approved the final manuscript.
